# 16S rRNA amplicon sequencing of seamount sediment microbial communities in South China Sea

**DOI:** 10.1128/mra.00678-25

**Published:** 2025-09-24

**Authors:** Haizhou Li, Huaiyang Zhou

**Affiliations:** 1 East China Sea Fisheries Research Institute, Chinese Academy of Fishery Sciences, Shanghai, China; 2 State Key Laboratory of Marine Geology, Tongji University 12476, Shanghai, China; Montana State University, Bozeman, Montana, USA

**Keywords:** South China Sea, seamount

## Abstract

Information about the microbiota in deep-sea seamount sediments is important because the microbiota and their activities in sediments affect deep-sea ecosystems. To evaluate deep-sea seamount microbial diversity, we performed 16S rRNA gene amplicon sequencing on sediment samples from 10 stations in the South China Sea.

## ANNOUNCEMENT

**Fig 1:**
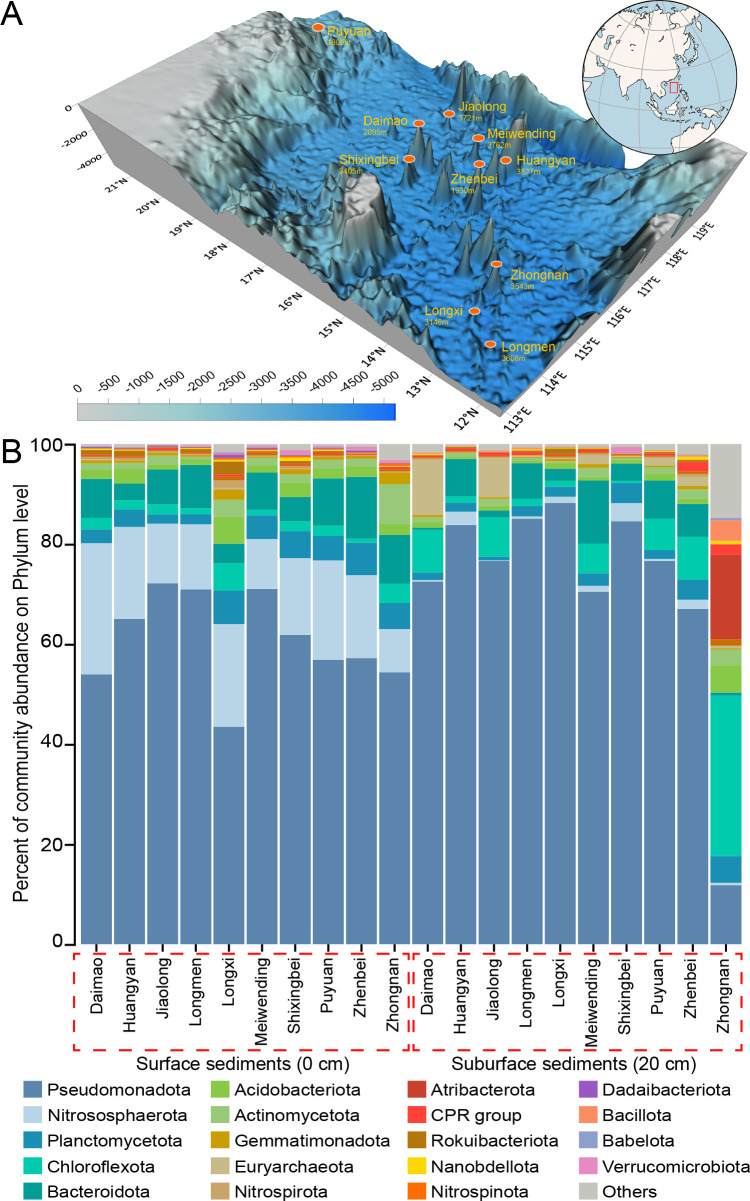
(**A**) Location of the South China Sea seamounts. The map shows the 10 seamount sampling locations and the bathymetry of the study area. This figure is reproduced from reference 2. (**B**) Column chart showing the relative abundance of the top 20 microbial phyla at each sampling station.

Although there are approximately 25 million seamounts in the ocean, surprisingly little is known about seamount microbial ecology (1, 2). The South China Sea (SCS) is the world’s largest semi-enclosed sea, which has a unique landscape with numerous basaltic seamounts in its basin (3). During the R/V Tan Kah Kee 1083 expedition (April to May 2018) in the SCS, the remotely operated platform for ocean science (ROPOS) was used to collect seamount sediment samples with a manipulated push corer on the summit of 10 seamounts (Fig. 1, Table 1). In this study, 16S rRNA gene amplicon sequencing was used to explore the diversity of microbial communities in SCS seamount sediment samples.

**TABLE 1:** Summary of sediment samples and sequencing results

Sample	Latitude	Longitude	Water depth (m)	No. of raw sequencing read		NCBI SRA accession no
Daimao	17°38.1296′N	117°6.4345′E	2,095 m	Surface	70,869	SRR14527283
				Subsurface	56,927	SRR14527282
Huangyan	15°17.2723′N	117°32.9357′E	3,827 m	Surface	50,316	SRR14527271
				Subsurface	72,279	SRR14527270
Jiaolong	17°32.181′N	117°44.3618′E	3,721 m	Surface	62,229	SRR14527269
				Subsurface	69,500	SRR14527268
Longmen	12°31.5517′N	113°41.534′E	3,608 m	Surface	63,288	SRR14527267
				Subsurface	72,272	SRR14527266
Longxi	13°7.6202′N	114°28.7122′E	3,146 m	Surface	71,703	SRR14527265
				Subsurface	44,547	SRR14527264
Meiwending	16°53.1704′N	118°0.4547′E	3,762 m	Surface	56,617	SRR14527281
				Subsurface	50,194	SRR14527280
Shixingbei	16°34.9017′N	116°24.0945′E	3,405 m	Surface	69,486	SRR14527277
				Subsurface	61,067	SRR14527276
Puyuan	21°8.0308′N	119°12.4658′E	1,805 m	Surface	56,984	SRR14527279
				Subsurface	71,331	SRR14527278
Zhenbei	14°59.8583′N	116°30.3167E	1,930 m	Surface	73,924	SRR14527275
				Subsurface	69,319	SRR14527274
Zhongnan	13°53.1921′N	115°19.8736′E	3,543 m	Surface	44,458	SRR14527273
				Subsurface	71,686	SRR14527272

The inner part of the push corer samples was collected to reduce contamination from the sampling process. The samples were stored at −80°C immediately after sampling. Surface sediments and subsurface (20 cm in depth) were used for amplicon sequencing following the Earth Microbiome Project (EMP) protocol (4). Total DNA was extracted using the E.Z.N.A. Soil DNA kit (Omega Bio-tek, Norcross, GA, USA). The 16S rRNA gene was amplified by PCR using universal bacterial and archaeal primers 515F and 806R (FWD: GTGYCAGCMGCCGCGGTAA; REV: GGACTACNVGGGGTWTCTAAT) (5). Each sample was amplified in triplicate in a 50 µL reaction under the following conditions: 30 cycles of denaturation at 94°C for 30 s, annealing at 55°C for 30 s, and extension at 72°C for 30 s, with a final extension at 72°C for 10 min. PCR products from each sample were pooled and purified using a QIAquick PCR Purification Kit (Qiagen) and quantified using a NanoDrop 1000 spectrophotometer (Thermo Scientific, USA). Purified amplicons were pooled in equimolar amounts and paired-end sequenced (2  ×  300 bp) on an Illumina MiSeq platform (Illumina, San Diego, CA, USA) (6). Raw fastq files were demultiplexed, quality filtered by Trimmomatic (v.0.39) (7), and merged by FLASH (v.1.2.11) (8). Operational taxonomic units (OTUs) were clustered with a 97% similarity cutoff using UPARSE (v.7.1) (9). Chimeric sequences were identified and removed using UCHIME (v.4.2) (10). Taxonomic assignments were performed using the SILVA database (v.138) (11). Default parameters were used except where otherwise noted.

A total of 6,391,730 16S rRNA gene amplicon sequences were generated and clustered into 16,051 OTUs. Here, we clustered merged reads into OTUs rather than inferring amplicon sequence variants to maintain compatibility with widely used EMP-style data sets. A range of 372 to 5,106 Chao1 OTUs and a Shannon index of 2.38 to 6.06 were observed in all the seamount systems. Eighty-eight microbial phyla and 1,453 genera were identified in the seamount samples. Pseudomonadota, Chloroflexota, and Actinomycetota were the most commonly identified phyla in all the sediment samples, and their total relative abundance accounted for approximately 63.84% (Fig. 1). The data in this study provide important information about the seamount microbial communities in deep-sea environments.

## Data Availability

16S rRNA amplicon DNA sequences from this study have been uploaded to the NCBI Sequence Read Archive (SRA) under accession number PRJNA729633.
